# The Diagnostic Accuracy of SARS-CoV-2 Nasal Rapid Antigen Self-Test: A Systematic Review and Meta-Analysis

**DOI:** 10.3390/life13020281

**Published:** 2023-01-19

**Authors:** Eleni Karlafti, Dimitrios Tsavdaris, Evangelia Kotzakioulafi, Georgia Kaiafa, Christos Savopoulos, Smaro Netta, Antonios Michalopoulos, Daniel Paramythiotis

**Affiliations:** 1 Emergency Department, University General Hospital of Thessaloniki AHEPA, Aristotle University of Thessaloniki, 54636 Thessaloniki, Greece; 2 1st Propaedeutic Department of Internal Medicine, AHEPA University General Hospital, Aristotle University of Thessaloniki, 54636 Thessaloniki, Greece; 3 First Propaedeutic Surgery Department, University General Hospital of Thessaloniki AHEPA, 54636 Thessaloniki, Greece

**Keywords:** diagnostic accuracy, SARS-CoV-2, antigen self-test, rt-PCR

## Abstract

Introduction: Severe acute respiratory syndrome coronavirus 2 (SARS-CoV-2) is the cause of coronavirus disease 2019 (COVID-19), a disease that quickly spread into a pandemic. As such, management of the COVID-19 pandemic is deemed necessary, and it can be achieved by using reliable diagnostic tests for SARS-CoV-2. The gold standard for the diagnosis of SARS-CoV-2 is a molecular detection test using the reverse transcription polymerase chain reaction technique (rt-PCR), which is characterized by various disadvantages in contrast with the self-taken nasal rapid antigen tests that produce results faster, have lower costs and do not require specialized personnel. Therefore, the usefulness of self-taken rapid antigen tests is indisputable in disease management, facilitating both the health system and the examinees. Our systematic review aims to access the diagnostic accuracy of the self-taken nasal rapid antigen tests. Methods: This systematic review was conducted following the Preferred Reporting Items for Systematic Reviews and Meta-Analyses (PRISMA) guidelines, and the Quality Assessment of Diagnostic Accuracy Studies 2 (QUADAS-2) tool was used to assess the risk of bias in the included studies. All the studies included in this systematic review were found after searching the two databases, Scopus and PubΜed. All but original articles were excluded from this systematic review, while all the studies concerning self-taken rapid antigen tests with a nasal sample and using rt-PCR as a reference test were included. Meta-analysis results and plots were obtained using RevMan software and the MetaDTA website. Results: All 22 studies included in this meta-analysis demonstrated a specificity of self-taken rapid antigen tests greater than 98%, which exceeds the minimum required yield for the diagnosis of SARS-CoV-2, according to the WHO. Notwithstanding, the sensitivity varies (from 40% to 98.7%), which makes them in some cases unsuitable for the diagnosis of positive cases. In the majority of the studies, the minimum required performance set by the WHO was achieved, which is 80% compared with rt-PCR tests. The pooled sensitivity of self-taken nasal rapid antigen tests was calculated as 91.1% and the pooled specificity was 99.5%. Conclusions: In conclusion, self-taken nasal rapid antigen tests have many advantages over rt-PCR tests, such as those related to the rapid reading of the results and their low cost. They also have considerable specificity and some self-taken rapid antigen test kits also have remarkable sensitivity. Consequently, self-taken rapid antigen tests have a wide range of utility but are not able to completely replace rt-PCR tests.

## 1. Introduction

In December 2019 in Wuhan, China identified the first known case of coronavirus disease 2019 (COVID-19), which quickly spread around the world. This highly contagious disease is caused by the SARS-CoV-2 virus [1] and spreads through droplet inhalation and direct contact with mucous membranes [2,3]. The majority of cases do not require hospitalization, as their severity is mild–moderate. The most common symptoms of the disease are fatigue, dry cough and fever, and it takes an average of 5 to 7 days for these symptoms to appear. In some cases, severe symptoms may occur and hospitalization and even admission to the ICU may be required. The prevention of COVID-19 spread and epidemic management depends on timely diagnoses [1,2,3,4,5].

Furthermore, the primary diagnostic test able to detect COVID-19 is the reverse transcription polymerase chain reaction (rt-PCR) test. This was the first test available to the public and able to identify SARS-CoV-2 virus molecules. It is a molecular test that analyzes an upper respiratory sample, detecting genetic material (ribonucleic acid or RNA) of SARS-CoV-2. The sample is collected with a sterile swab in a manner similar to that of the rapid antigen test or with a saliva tube. An rt-PCR test is more accurate, time-consuming and expensive compared with a rapid antigen test. Another disadvantage of rt-PCR tests is the necessity of specialized personnel and specific technical equipment. According to the above, the rt-PCR test becomes unsuitable in certain circumstances, such as emergency situations or in underdeveloped countries [6,7].

In addition, prevention of virus spread could be achieved through intensive screening and timely identification of virus-infected individuals. Screening can be managed by using rapid antigen SARS-CoV-2 tests, which include those with a nasal sample, a nasopharyngeal sample or a saliva sample. However, the most useful of them are the self-taken rapid antigen tests (using nasal samples) because of their low cost, quick results and, at the same time, the needlessness for specialized personnel or special experience [7]. A self-taken rapid antigen test comprises a rapid chromatographic immunoassay for the qualitative detection of specific antigens of SARS-CoV-2. A self-taken rapid antigen test kit contains a sterile swab that is inserted into both nostrils and rotated. Then, the swab is placed into an extraction buffer tube and stirred. A few drops (three to five, depending on the kit) of the extracted sample are added to the specimen well of the test device. The results are visible within a short period of time and must be read within the first half hour [7,8].

Thus, a self-taken rapid antigen test has many advantages over an rt-PCR test regarding its utility and practicality. The major question is whether self-taken rapid antigen test kits are reliable enough to replace the use of rt-PCR tests, at least when carrying out the latter is not feasible. For this reason, various studies have been conducted with the aim of finding the diagnostic accuracy of these tests, taking into consideration that for the diagnosis of SARS-CoV-2, according to the World Health Organization (WHO), a self-taken rapid antigen test kit needs to meet a minimum performance requirement of at least 80% sensitivity and 97% specificity compared with a nucleic acid amplification test (NAAT) used as a reference assay [9].

The aim of this systematic review is to assess the diagnostic accuracy of the self-taken rapid antigen test, which offers easier and more tolerable sampling than the nasopharyngeal sampling, using as a reference standard the rt-PCR test, which is the most reliable option to date.

## 2. Materials and Methods

### 2.1. Study Protocol and Guidelines

This systematic review was written according to the Preferred Reporting Items for Systematic Reviews and Meta-Analyses (PRISMA) guidelines [10]. This systematic review was registered in the Open Science Framework (OSF). This registration can be found using the link osf.io/fkyvb and with digital object identifier DOI 10.17605/OSF.IO/FKYVB.

### 2.2. Eligibility Criteria

The inclusion criteria were the following:

- Use of self-taken rapid antigen test as an index test;
- Use of rt-PCR as a reference standard;
- Presence of the sensitivity and specificity of rapid antigen test;
- Use of nasal specimen;
- English language.

The exclusion criteria were the following:

- Duplicate articles;
- Original investigation;
- Reviews;
- Editorials;
- Letters;
- Comments;
- Meta-analysis articles.

### 2.3. Information Sources, Search Strategy and Selection Process

The search was carried out in the online databases PubMed and Scopus using the keywords ‘COVID-19’, ‘self-rapid test’ and ‘diagnostic accuracy’. The only filter used was the writing of the article in the English language. The search was conducted in October 2022 and the time limit was from November 2019 (emergence of SARS-CoV-2 in China) until October 2022. The selection process was carried out by two independent reviewers (D.T. and E.Kar.) who assessed the studies’ eligibility for inclusion in this systematic review using the title, abstract and full-text evaluation. Any disagreement was solved by a third reviewer (E.K.).

### 2.4. Data Collection Process and Data Items

The reviewers selected as many data as were considered useful for conducting the systematic review without the use of any automation tools. The main characteristics of each study [11,12,13,14,15,16,17,18,19,20,21,22,23,24,25,26,27,28,29,30,31,32] are included in Table 1. These characteristics were, more specifically, the identity of each study (author and the year of publishing), the location, the total participants, the type of study, the self-taken rapid antigen test kit that was used, and the sensitivity, specificity, negative predictive value, positive predictive value and accuracy of each self-taken rapid antigen test kit. Any diagnostic parameter not already calculated was calculated using the true positive (TP), true negative (TN), false positive (FP) and false negative (FN) values using RevMan 5.4 software. The abbreviation TP stands for true positive, namely, that the test result correctly indicates the presence of disease, whereas TN stands for true negative, namely, that the test result correctly indicates the absence of disease. Correspondingly, FP stands for false positive, denoting that the test result wrongly indicates the presence of disease, and FN stands for false negative, denoting that the test result wrongly indicates the absence of disease.

### 2.5. Study Risk of Bias Assessment

The Quality Assessment of Diagnostic Accuracy Studies 2 (QUADAS-2) tool was used to assess the risk of bias in the included studies, consisting of four key domains (patient selection, index test, reference standard, and flow and timing) [26,27].

### 2.6. Statistical Analysis

ReviewManager 5.4 software and also the online website MetaDTA were used to quantitatively synthesize the results of each study using TP, TN, FP and FN values. By using the forest plots, the overall results are illustrated.

## 3. Results

### 3.1. Study Selection

**Figure 1:**
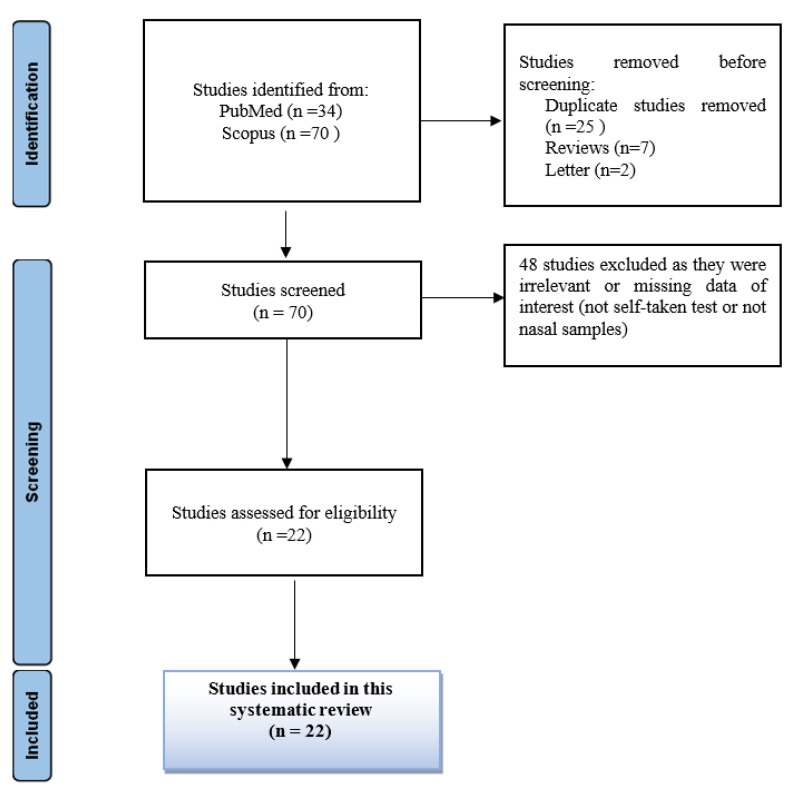
PRISMA 2020 flow diagram. This diagram illustrates the process followed for the collection and selection of studies used in our systematic review.

A total of 104 results were found after searching the keywords in the PubMed and Scopus databases. Of these, 34 were not included as they concerned reviews, letters or were duplicate studies. Of the remaining 70 studies, 48 were removed as they did not meet the inclusion criteria for this systematic review. Finally, 22 studies were included. The screening process of studies selected for inclusion in this systematic review is illustrated in the PRISMA 2020 flow chart (Figure 1). All selection stages from the initial stage to the final stage are depicted [28,29].

### 3.2. Study Characteristics

A total of 22,160 people participated in the studies, including those who tested positive or negative for COVID-19 according to the results of rt-PCR tests and those who were symptomatic or asymptomatic. Of the 22,160 people who participated in these studies, 17,045 relate to control for the diagnostic accuracy of the rapid antigen self-taken test. The twenty-two included studies were conducted in twelve different countries (Malaysia, U.K., U.S.A., Germany, Canada, the Netherlands, India, Denmark, France, Greece, Austria and Israel), and twenty-three different self-taken rapid antigen test kits were used in these studies. All detailed information is shown in Table 1. All statistical characteristics were calculated with ReviewManager 5.4 software using the following statistical formulas [30]:

- Sensitivity = TP/(TP + FN);
- Specificity = TN/(TN + FP);
- Negative predictive value (NPV) = TN/(TN + FN);
- Positive predictive value (PPV) = TP/(TP + FP);
- Accuracy = (TP + TN)/(TP + TN + FP + FN).

Of the 22 studies reviewed, 9 [11,12,13,14,18,25,35,37,39] used self-taken rapid antigen tests approved by the WHO for professional use [31], which are the STANDARD-Q COVID-19 Ag Test, Panbio COVID-19 Ag Rapid Test, LumiraDx SARS-CoV-2 Ag Test, Abbott Panbio COVID-19 Ag Rapid Test and SARS-CoV-2 Antigen Rapid Test (Flowflex).

**Figure 2:**
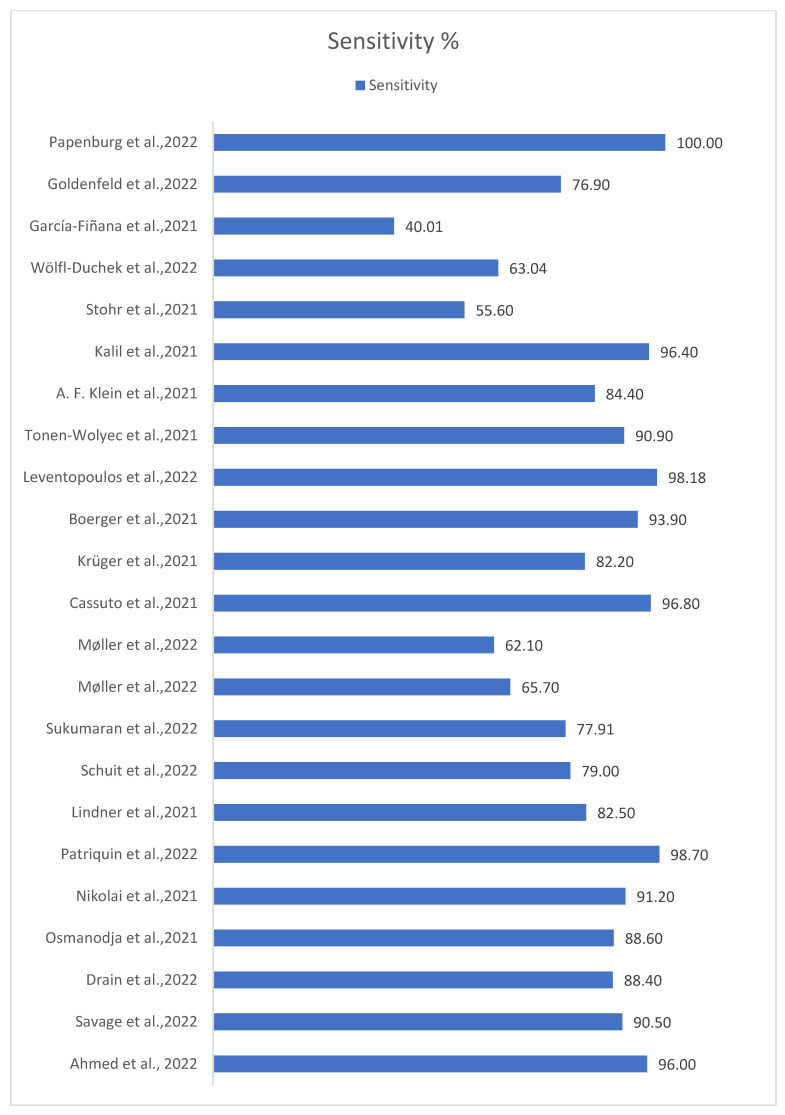
Sensitivity percentages of included studies. The term sensitivity (true positive rate) refers to the probability of a positive test, conditioned on it truly being positive [11,12,13,14,15,16,17,18,19,20,21,22,23,24,25,33,34,35,36,37,38,39].

**Figure 3:**
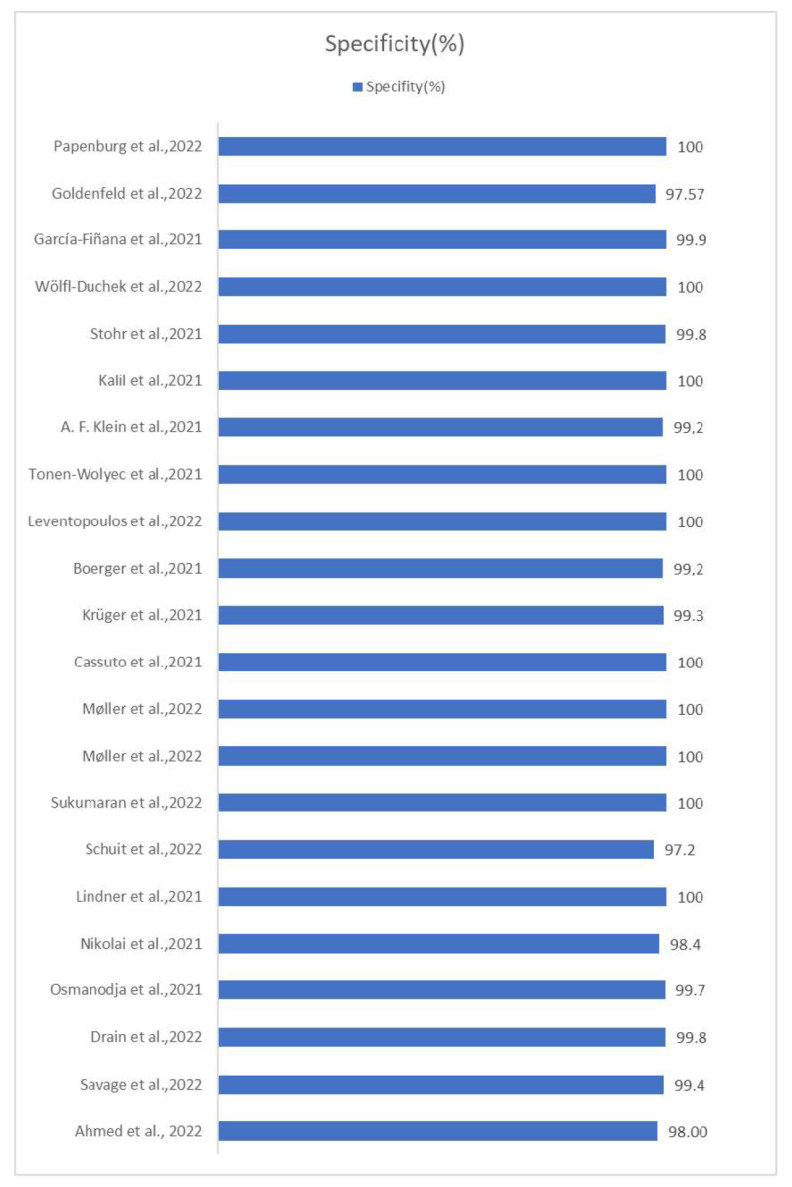
Percentage specificity of each study. The term specificity (true negative rate) refers to the probability of a negative test, conditioned on it truly being negative. Patriquin et al.’s specificity was not estimable [11,13,14,15,16,17,18,19,20,21,22,23,24,25,33,34,35,36,37,38,39].

The highest sensitivity was found in Patriquin et al.’s 2022 study with a sensitivity of 98.7% [12], while the lowest was found in Garcia-Finana et al.’s 2021 study with a sensitivity of just 40% [24]. Regarding specificity, 14 of the 22 studies showed 100% specificity, a result that emphasizes the reliability of the self-taken rapid antigen tests in terms of true negative tests, and this is due to the very low rates of false positive tests in each of these studies. In one of these twenty-two studies [12], the specificity could not be calculated because the selection of candidates was based on the existence of a positive Ag-RDT NP swab. The sensitivity and specificity of each study are illustrated in Figure 2 and Figure 3 respectively.

Moreover, after the first outbreak of the SARS-CoV-2 pandemic, various mutations of the virus prevailed, such as Alpha and Omicron; however, self-taken rapid antigen tests are able to diagnose all existing mutations up to now, [32] except in the case of the B.1.1.7 variant of concern mutation (VOC), which has been found to affect the performance of self-taken rapid antigen tests by increasing false negative results [32].

### 3.3. Meta-Analysis

**Figure 4:**
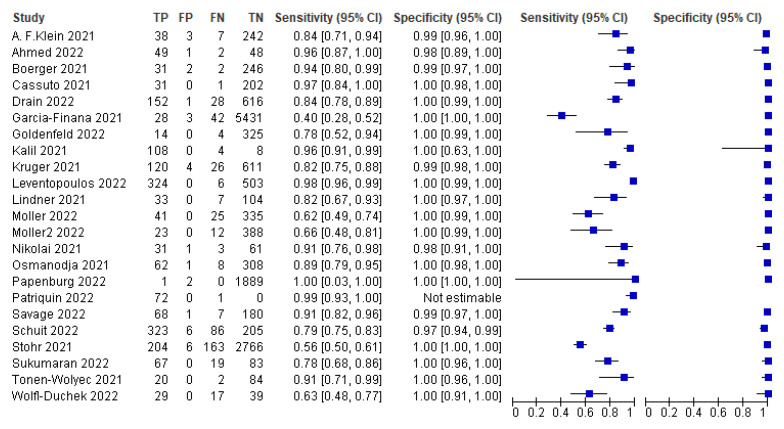
Forest plots of pooled sensitivity and specificity. TP, FP, FN and TN values for each study as well as calculated sensitivities and specificities are depicted. Patriquin et al.’s specificity was not estimable [11,12,13,14,15,16,17,18,19,20,21,22,23,24,25,33,34,35,36,37,38,39].

**Figure 5:**
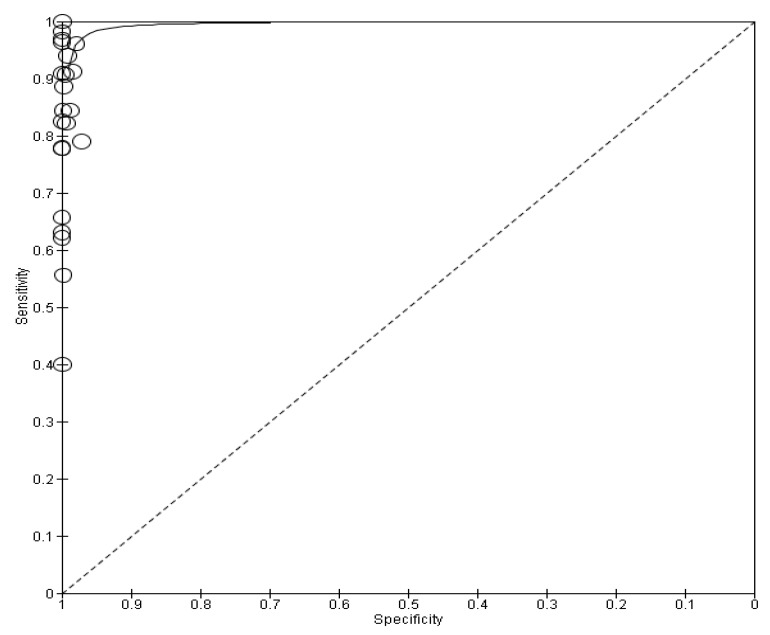
SROC plot. The circles depict the sensitivity of each rapid antigen test kit and the dashed line depicts the bisector of the plot [11,12,13,14,15,16,17,18,19,20,21,22,23,24,25,33,34,35,36,37,38,39].

The pooled sensitivity of self-taken rapid antigen tests as well as the pooled specificity were calculated using the online website MetaDTA. The meta-analysis demonstrated a pooled sensitivity of 91.1% (95% CI: 84.3–95.1) and a pooled specificity of 99.5% (95% CI: 99–99.8), as shown in Table 2. Below is the forest plot with the values of TP, TN, FP and FN (Figure 4) as well as the SROC plot (Figure 5), which were generated using RevMan software. In contrast, RT-PCR, which is currently the gold-standard technique, has a sensitivity of greater than 97% [40,41], which reaches up to 100% for many rt-PCR kits [41].

These results confirm that self-taken rapid antigen tests can be used as an alternative to rt-PCR tests as they are reliable enough to make a diagnosis of SARS-CoV-2 or reject it. However, they are not superior to rt-PCR tests, which are the primary tests for the diagnosis of SARS-CoV-2.

**Figure 6:**
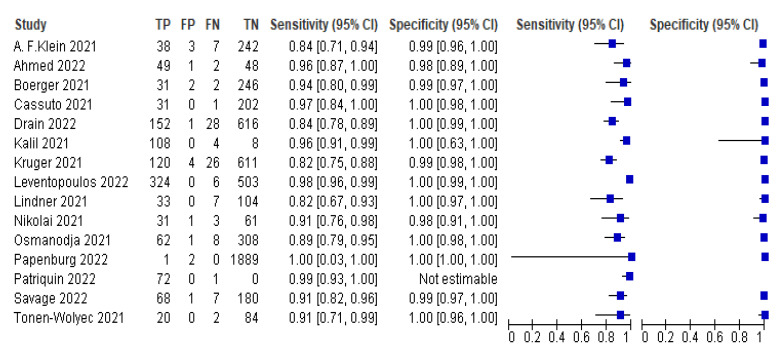
Forest plots of pooled sensitivity and specificity of the subgroup. TP, FP, FN and TN values for each study as well as calculated sensitivities and specificities are depicted. Patriquin et al.‘s specificity was not estimable. This subgroup includes only the 15 studies that used rapid antigen test kits that achieve the minimum acceptable performance according to WHO [11,12,13,17,18,19,20,21,33,34,35,36,37,38,39].

**Figure 7:**
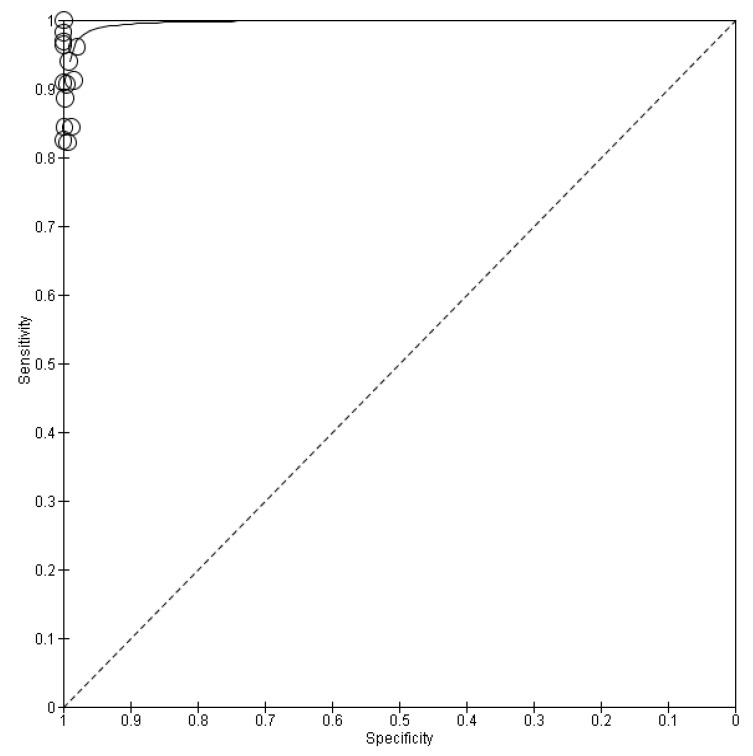
SROC plot of the subgroup. The circles depict the sensitivity of each rapid antigen test kit and the dashed line depicts the bisector of the plot [11,12,13,17,18,19,20,21,33,34,35,36,37,38,39].

Of the 22 studies included in this meta-analysis, 7 used rapid antigen test kits that did not achieve the minimum acceptable performance according to the WHO. For this reason and because these rapid antigen test kits cannot be used reliably due to their performance, the separation of these seven studies from the pooled sensitivity and the pooled specificity was carried out [14,15,16,22,23,24,25]. More precisely, this subgroup includes only the 15 studies that used rapid antigen test kits that achieve the minimum acceptable performance according to the WHO. Below is the forest plot with the values of TP, TN, FP and FN (Figure 6) as well as the SROC plot (Figure 7). Thus, the pooled sensitivity and pooled specificity were then calculated to be 98.8% and 97.4%, respectively, ) as shown in Table 3.

These results show an even higher sensitivity for the rapid antigen test kits, further enhancing their diagnostic accuracy and their potential for use in daily practice. These results emphasize that the correct selection of self-taken rapid antigen test kits (that is, with performances higher than the minimum performances according to the WHO) can give us results that are quite close to those of rt-PCR tests and, therefore, can be considered reliable enough. However, these results depend on many factors that must be taken into account to evaluate the results (the sampling techniques [42], the storage and transportation of the self-taken rapid antigen test kits [43] and viral load [44]).

QUADAS-2 was used to assess the quality of the research. Each domain was assessed in terms of risk of bias, and the first three domains were also assessed in terms of concerns regarding applicability. Using this tool checks for bias and ensures the transparency of this systematic review [26,27]. The results of the application of this tool to the studies analyzed are shown in the table (Table 4) below and can be low, high or unclear for each category.

## 4. Discussion

This systematic review revealed that self-taken rapid antigen tests have a very high specificity, as all the included studies indicated a specificity greater than or equal to 98% in comparison with the results of the rt-PCR tests. However, a wide range of variation in sensitivity was found, which ranged from 40% to 98.7%, with the majority of studies (14 of them) showing values greater than 80%, which is considered the minimum performance by the WHO for the diagnosis of COVID-19 [9]. The terms sensitivity (true positive rate) and specificity (true negative rate) refer to the probability of a positive test, conditioned on truly being positive, and to the probability of a negative test, conditioned on truly being negative [30]. Therefore, self-taken rapid antigen tests are capable enough to diagnose a truly negative case but fall short of diagnosing positive cases. However, they can replace the use of rt-PCR tests when the latter is not available.

Furthermore, this meta-analysis shows that self-taken rapid antigen tests are quite capable of diagnosing SARS-CoV-2 as they scored a pooled sensitivity of 91.1% and a pooled specificity of 99.5%, while the subgroup with self-taken rapid antigen test kits with performances higher than the minimum performances according to the WHO scored a pooled sensitivity of 98.8% and a pooled specificity of 97.4%.

Moreover, the results of the self-taken (nasal) rapid antigen tests also depend directly on the sampling technique [42] and on the storage and transportation of the self-taken rapid antigen test kits [43]. Regarding the technique, the study of Tonen-Woleyc et al., 2021, revealed that the majority of participants (94.4%) understood the sample collection instructions [20], and in another study (Jing et al., 2022) 255 of 264 (96.6%) participants were able to perform the test successfully [42]. As a result, in many of these studies, the sensitivity and specificity were found to be the same between healthcare providers and non-healthcare providers [11,17,18,21,23,37]. Indeed, Savage et al., 2022, revealed that the sensitivity of self-taken rapid antigen tests was higher (90.5%) compared with professional-taken tests (78.3%) [34]. Consequently, taking the samples from the examinees themselves does not seem to particularly affect the rapid antigen test results as long as they have been adequately informed about the correct sampling technique.

Furthermore, the storage and transportation of self-taken rapid antigen test kits can also affect their diagnostic accuracy. The repeated freezing and thawing process of the self-taken rapid antigen test kits, which takes place during their transport, can affect the contained proteins, significantly reducing their diagnostic accuracy [45,46]. The above mainly affects countries that do not manufacture self-taken rapid antigen test kits but instead import them, such as African and Asian countries. Indeed, in these countries, self-taken rapid antigen tests are preferred more than rt-PCR tests due to their lower cost. Thus, it is considered necessary to find the appropriate storage and transport conditions in order to eliminate this factor that reduces the performance of self-taken rapid antigen tests [43].

Nevertheless, the viral load seems to significantly affect the results of self-taken rapid antigen tests [44] and contributes an important factor for the low sensitivity of the tests. Specifically, the sensitivity was found to be higher in patients with higher viral loads and also in symptomatic patients. The majority of these studies indicate the greater sensitivity of the self-taken rapid antigen tests in symptomatic patients where the viral load is higher [11,16,18,20,21,24]. Nikolai et al. [11] suggest that blowing the nose once before sampling could increase the viral load in the nostrils. Therefore, is recommended to repeat the test after at least 48 h [24] or after the appearance of symptoms [11,16,18,20,21,24].

In addition, among the rapid antigen tests that were used in each of the studies, those that did not exceed the minimum performance according to the WHO [9] are the following: FlowFlex, MPBio, Clinitest, AG-Q COVID-19 N-Ag rapid antigen test by Agappe, COVID-19 rapid antigen tests from DNA Diagnostic A/S, the RAT from Hangzhou Immuno Biotech Co Ltd., BD-RDT, Medomics SARS-CoV-2 antigen test device, Innova LFT and the Nowcheck rapid antigen tests with scores of 79%, 77.9%, 65.7%, 62.1%, 55.6%, 63%, 40%, and 76.9%, respectively [14,15,16,22,23,24,25]. All self-taken rapid antigen test kits exceed the minimum performance according to the WHO in terms of specificity, i.e., more than 97%.

Furthermore, most of the studies mentioned in this meta-analysis [11,12,13,14,15,16,17,18,20,21,22,23,25,33,35,36,38,39] also used nasopharyngeal samples. Rapid antigen tests using nasopharyngeal samples are considered more reliable compared with nasal swab use due to the higher concentration of the virus [47]. However, a rapid antigen test’s performance by collecting a nasopharyngeal sample requires specialized personnel, as well as the appropriate equipment, and the procedure is quite inconvenient and even painful for the examinees. This need for specialized personnel for sampling burdens the health system with further work and expenses. On the other hand, in self-taken rapid antigen tests, there is no need for the patient to contact healthcare workers and, consequently, the possible virus spread is avoided, not only to healthcare providers but also, through them, to other patients. Conclusively, rapid antigen tests using nasopharyngeal samples are considerably more expensive, time-consuming and burdensome for the healthcare system compared with self-taken rapid antigen tests, while they can still pose a risk to public health [34].

Furthermore, in a meta-analysis by Brümmer et al. regarding the diagnostic accuracy of self-taken rapid antigen tests, [48] found 71.2% sensitivity and 98.9% specificity. Although the specificity calculated in this study is similar to the results of our study, the sensitivity differs by a significant percentage (19.9%). This difference may be due to any of the factors affecting sensitivity. This meta-analysis lags behind ours in terms of date, as it was published in August 2021 and 16 of the studies [11,12,14,15,16,18,19,20,21,23,25,33,34,35,37,38] we have included were published after the publication of the mentioned meta-analysis. Moreover, the meta-analysis by Brümmer et al. concerns all types of rapid antigen tests (using nasal samples, nasopharyngeal samples and saliva samples) in contrast with ours, which calculates the diagnostic accuracy with nasal samples.

Moreover, a meta-analysis by Dinnes et al. in 2022 [49] concerning the diagnostic accuracy of self-taken rapid antigen tests categorizes its results according to the presence or absence of symptoms and then, by the time of taking the sample in relation to the time of appearance of symptoms, i.e., if the test was performed in the first or second week of symptoms appearance. The results of this study showed a similar specificity to the specificity we calculated in our meta-analysis. However, sensitivity differs between symptomatic patients (estimated at 73%) and asymptomatic patients (estimated at 54.7%). When a test was performed in the first week of symptoms, the sensitivity increased significantly to 81%, whereas when it was performed during the second week, it decreased again to 54%. This meta-analysis also lags behind ours in terms of its publication date because four of the studies [14,25,35,38] we have included were published after its publication. Additionally, this meta-analysis, as with the previous one, examines the diagnostic accuracy of all types of rapid antigen tests in contrast with ours.

Furthermore, due to low cost, rapid results, and an easy sampling and testing method, the use of self-taken rapid antigen tests is popular in various asymptomatic groups in terms of prevention. More specifically, systematic repeated tests for SARS-CoV-2 diagnoses for some population groups, such as students and education staff, healthcare workers, and employees of large companies or services, is a tactic due to virus spread management. There are not enough studies confirming the reliability of this practice. However, Winkel et al. reveal that a weekly self-taken rapid antigen test can identify 90% of patients with pre-symptomatic or early infection [50], while Love et al. indicate the detection of 68% of asymptomatic patients [51]. Therefore, by establishing regular repeated tests in asymptomatic patients, a large percentage of the carriers of the virus can be detected and isolated.

Further to this, the performance of self-taken rapid antigen tests in children and adults had little to no difference when these groups were compared. This is due to the similar viral load between the two age groups. However, any differences may be due to adequate sampling or even the individual characteristics of the examinees [52,53]. In addition, an incorrect sampling technique can increase the chance of error and is more likely to occur in young children. Moreover, regarding disease manifestation, there are less symptoms in children compared with adults. On the other hand, the sensitivity of self-taken rapid antigen tests in symptomatic patients is increased, but it seems that the result is not significantly affected in all age groups [53].

Nevertheless, rt-PCR tests are not only characterized by their disadvantages (high cost, time-consuming and need for specialized personnel) compared with self-taken rapid antigen tests, but also by the doubt about whether individuals with positive test results could transmit the virus [54]. This doubt arises due to the high sensitivity of rt-PCR tests, such that even a very small concentration of the virus can give a positive test result [55]. Furthermore, the genetic material of the virus remains in the human body, even after the inactivation of the virus, for some period of time. Thus, individuals who have recovered and are not capable of transmitting the virus may produce a positive test result [55]. In contrast, self-taken rapid antigen tests are more sensitive to high viral loads, providing an advantage in screening efforts.

Further investigations are necessary to improve the self-taken rapid antigen test technique in order to optimize its utility. Simultaneously, further investigations could also be designed to develop and improve the diagnostic accuracy of rapid antigen tests that use saliva samples because this technique is even easier and less painful for the examiner. It also needs to be clarified whether any mutations in the nucleotide capsid of the virus can affect the performance of self-taken rapid antigen tests. So far, it has been found that the tests were not affected by the mutations that have occurred in the nucleotide capsid of the virus [14]. Lastly, further studies may also be conducted to test the use of self-taken rapid antigen tests regarding other viruses.

This study has limitations. One limitation is the lack of information about the accuracy of the self-taken rapid antigen tests in the included studies. The small availability of studies on the subject, however, gives this systematic review strength, as it facilitates the synthesis of the results while simultaneously reducing the possibility of error.

## 5. Conclusions

In conclusion, self-taken rapid antigen tests have many advantages over rt-PCR tests, such as those related to the rapid reading of results and their low cost. The specificity of self-taken rapid antigen tests approaches the rt-PCR specificity, while their sensitivity is remarkable. Furthermore, the sensitivity of self-taken rapid antigen tests increases significantly in symptomatic patients and in patients examined during the first week of symptoms, making the self-taken rapid antigen tests even more reliable. Moreover, using the correct technique is associated with the performance of self-taken rapid antigen tests, so it is recommended that examinees be fully informed about the correct procedure of sampling. Consequently, self-taken rapid antigen tests have a wide range of utility, but are not able to completely replace rt-PCR tests. Until the weakness of self-taken rapid antigen tests regarding their low sensitivity is addressed, rt-PCR tests remain the gold standard for the diagnosis of SARS-CoV-2.

## Figures and Tables

**Table 1:** Major characteristics of included studies. These characteristics include the identity of each study (author and the year of publishing), the location, the total participants, the type of study, the self-taken rapid antigen test kit that was used, and the sensitivity, specificity, negative predictive value, positive predictive value and accuracy of each self-taken rapid antigen test kit. Abbreviations: NM—not mentioned.

Study ID	Location	Total Subjects (% Female/Median Age)	Type of Research	Rat Kit	Sensitivity %	Specificity %	Negative Predictive Value %	Positive Predictive Value %	Accuracy %
Ahmed et al., 2022 [33]	Kelantan, Malaysia	157 (42, 67/NM)	Cross-sectional study	Κit ProDetect, Medical Innovation Ventures Sdn Bhd, Malaysia	96.00	98.00	96.00	98.00	97.00
Savage et al., 2022 [34]	Liverpool, U.K.	250 (58.3/40)	Prospective diagnostic accuracy evaluation	Covios COVID-19 Antigen Rapid Diagnostic test	90.5	99.4	96.1	98.5	96.9
Drain et al., 2022 [35]	King County, Washington, U.S.A.	802 (58.2/37.3)	Clinical diagnostic accuracy study	Ag Detect Rapid Self-Test (InBios International Inc) and BinaxNOWCOVID-19 Ag Card (Abbott Laboratories)	84.4	99.8	95.6	99.3	96.4
Osmanodja et al., 2021 [36]	Berlin, Germany	379 (NM/NM)	Prospective diagnostic accuracy study	Nasal swab for the Ag-RDT Dräger Antigen Test SARS-CoV-2 by Dräger Safety AG and Co. KGaA, Lübeck, Germany	88.6	99.7	99.3	98.5	99.4
Papenburg et al., 2022 [37]	Montreal, Canada	278 (43.9/43)	Cross-sectional study	Panbio COVID-19 Ag Rapid Test Device (Abbott Laboratories)	100	100	100	33.3	99.8
Nikolai et al., 2021 [11]	Berlin, Germany	228 (46.7/34.6)	Prospective diagnostic accuracy study	STANDARD Q COVID-19 Ag Test (SD Biosensor, Korea)	91.2	98.4	96.9	96.8	96.8
Patriquin et al., 2022 [12]	Nova Scotia, Canada	197 (NM/NM)	Comparative Study	Abbott Panbio COVID-19 Ag rapid test device (Abbott Rapid Diagnostics GmbH, Orlaweg, Germany)	98.7	NM	NM	88.8	NM
Lindner et al., 2021 [13]	Berlin, Germany	146 (51.4/35)	Prospective diagnostic accuracy study	STANDARD Q COVID-19 Ag Test (SD Biosensor, Inc. Gyeonggi-do, Korea)	82.5	100	99.1	91.4	95.1
Schuit et al., 2022 [14]	The Netherlands	6497 (NM/NM)	Prospective cross sectional diagnostic test accuracy	Flowflex (Acon Laboratories), MPBio (MP Biomedicals) and Clinitest (Siemens-Healthineers)	79	97.2	70.4	98.2	85.1
Sukumaran et al., 2022 [15]	Kerala, India	150 (NM/NM)	Validation study	AG-Q COVID-19 N-Ag rapid test kit by Agappe Diagnostics Limited.	77.91	100	77.11	100	87.33
Møller et al., 2022 [16]	Aarhus, Denmark	827 (50.5/NM)	Prospective diagnostic accuracy study	COVID-19 Antigen Detection Kit-DNA Diagnostic A/S, Risskov, Denmark and the SARS-CoV-2 Antigen Rapid Test-Hangzhou Immuno Biotech Co Ltd, Hangzhou, China)	65.7 and 62.1	100 and 100	97 and 93	100 and 100	97.1 and 93.7
Cassuto et al., 2021 [17]	Paris, France	239 (NM/NM)	Validation study	COVID-VIRO® Antigen test	96.8	100	99.5	100	99.5
Krüger et al., 2021 [18]	Heidelberg and Berlin, Germany.	761 (52/35)	Diagnostic accuracy study	LumiraDx™	82.2	99.3	99.8	45.8	99.6
Boerger et al., 2021 [19]	Rochester, Minnesota, U.S.A.	300 (NM/NM)	Prospective diagnostic accuracy study	Flocked MT swab (Copan Diagnostics, Murrieta, CA)	93.9	99.2	99.1	93.9	98.6
Leventopoulos et al., 2022 [38]	Athens, Greece	833 (46.7/32.94)	Prospective diagnostic accuracy study	Boson Rapid Antigen Test Card	98.18	100	98.2	100	99.28
Tonen-Wolyec et al., 2021 [20]	Paris, France	106 (62.4/40)	Prospective diagnostic accuracy study	Biosynex Antigen Self-Test COVID-19 AG+	90.9	100	97.6	100	98.1
Klein et al., 2021 [39]	Heidelberg, Germany	290 (52.4/42.7)	Prospective diagnostic accuracy study	Panbio™ Ag-RDT (distributed by Abbott)	84.4	99.2	99.8	88.1	96.5
Kalil et al., 2021 [21]	Kelantan, Malaysia	120 (54.2/NM)	Prospective diagnostic accuracy study	ProdetectTM COVID-19 Antigen rapid self-test	96.4	100	66.6	100	96.6
Stohr et al., 2021 [22]	Breda, the Netherlands	3201 (56.5/41)	Cross-sectional study	BD-RDT or Roche-RDT	55.6	99.8	94.4	97.1	94.6
Wölfl-Duchek et al., 2022 [23]	Vienna, Austria	132 (41.3/62.2)	Prospective diagnostic accuracy study	Medomics SARS-CoV-2 antigen test device (Jiangsu Medomics Medical Technology Co., China)	63.04	100	70.69	100	80.46
Garcia-Fiñana et al., 2021 [24]	Liverpool, U.K.	5869 (NM/NM)	Observational cohort study	Innova LFT	40	99.9	99.2	90.3	99.1
Goldenfeld et al., 2022 [25]	Ramat Gan, Israel	398 (NM/NM)	Prospective diagnostic accuracy study	Nowcheck RAT (Bionote, South Korea), SD RAT, Roche, SSS Australia) and Panbio RAT, Abbott Diagnostic, Jena	76.9	97.57	98.7	100	98.8

**Table 2:** Estimates.

Parameter	Estimate	2.5% CI	97.5% CI
Sensitivity	0.911	0.843	0.951
Specificity	0.995	0.990	0.998
False positive rate	0.005	0.002	0.010
Logit (sensitivity)	2.328	1.680	2.976
Logit (specificity)	5.359	4.583	6.136

**Table 3:** Estimates of the subgroup.

Parameter	Estimate	2.5% CI	97.5% CI
Sensitivity	0.988	0.962	0.996
Specificity	0.974	0.941	0.989
False positive rate	0.026	0.011	0.059
Logit(sensitivity)	4.436	3.241	5.631
Logit(specificity)	3.634	2.774	4.494

**Table 4:** Quality assessment results. Each domain is assessed in terms of risk of bias, and the first 3 domains are also assessed in terms of concerns regarding applicability. The letter L corresponds to low, H to high and U to unclear.

	Risk of Bias	Risk of Bias	Risk of Bias	Risk of Bias	Applicability Concerns	Applicability Concerns	Applicability Concerns
Studies	Patient Selection	Index Test(S)	Reference Standard	Flow And Timing	Patient Selection	Index Test(S)	Reference Standard
Ahmed et al., 2022 [33]	L	L	L	L	L	L	L
Savage et al., 2022 [34]	L	L	L	L	L	L	L
Drain et al., 2022 [35]	L	L	L	L	L	L	L
Osmanodja et al., 2021 [36]	L	L	L	L	L	L	L
Papenburg et al., 2022 [37]	L	L	L	U	L	L	L
Nikolai et al., 2021 [11]	L	L	L	L	L	L	L
Patriquin et al., 2022 [12]	L	L	L	U	L	L	L
Lindner et al., 2021 [13]	L	L	L	L	L	L	L
Schuit et al., 2022 [14]	L	L	L	H	L	L	L
Sukumaran et al., 2022 [15]	L	L	L	L	L	L	L
Møller et al., 2022 [16]	L	L	L	L	L	L	L
Cassuto et al., 2021 [17]	L	L	L	L	L	L	L
Krüger et al., 2021 [18]	L	L	L	L	L	L	L
Boerger et al., 2021 [19]	L	L	L	L	L	L	L
Leventopoulos et al., 2022 [38]	L	L	L	L	L	L	L
Tonen-Wolyec et al., 2021 [20]	L	L	L	L	L	L	L
Klein et al., 2021 [39]	L	L	L	L	L	L	L
Kalil et al., 2021 [21]	L	L	L	L	L	L	L
Stohr et al., 2021 [22]	L	L	L	L	L	L	L
Wölfl-Duchek et al., 2022 [23]	L	L	L	U	L	L	L
García-Fiñana et al., 2021 [24]	L	L	L	L	L	L	L
Goldenfeld et al., 2022 [25]	L	L	L	U	L	L	L
